# Impact of pharmacist services on economic, clinical, and humanistic outcome (ECHO) of South Asian patients: a systematic review

**DOI:** 10.1186/s40545-022-00431-1

**Published:** 2022-05-10

**Authors:** Sunil Shrestha, Rajeev Shrestha, Ali Ahmed, Binaya Sapkota, Asmita Priyadarshini Khatiwada, Christina Malini Christopher, Parbati Thapa, Bhuvan KC, Ali Qais Blebil, Saval Khanal, Vibhu Paudyal

**Affiliations:** 1 School of Pharmacy, Monash University Malaysia, Jalan Lagoon Selatan, 47500 Bandar Sunway, Selangor Malaysia; 2 Department of Pharmacy, District Hospital Lamjung, Besisahar, Province Gandaki Nepal; 3 Department of Pharmaceutical Sciences, Nobel College, Affiliated to Pokhara University, Kathmandu, Province Bagmati Nepal; 4 Department of Pharmaceutical and Health Service Research, Nepal Health Research and Innovation Foundation, Lalitpur, Province Bagmati Nepal; 5 Division of Health Sciences, Warwick Medical School, University of Warwick, Coventry, UK; 6 School of Pharmacy, College of Medical and Dental Sciences, University of Birmingham, Birmingham, UK

**Keywords:** Economic, Clinical and humanistic outcome (ECHO), Health-related quality of life (HRQOL), Pharmacist, South Asia

## Abstract

**Background:**

Pharmacists in high-income countries routinely provide efficient pharmacy or pharmaceutical care services that are known to improve clinical, economic, and humanistic outcomes (ECHO) of patients. However, pharmacy services in low- and middle-income countries, mainly South Asia, are still evolving and limited to providing traditional pharmacy services such as dispensing prescription medicines. This systematic review aims to assess and evaluate the impact of pharmacists’ services on the ECHO of patients in South Asian countries.

**Methods:**

We searched PubMed/Medline, Scopus, EMBASE, CINAHL, and Cochrane Library for relevant articles published from inception to 20th September 2021. Original studies (only randomised controlled trials) conducted in South Asian countries (published only in the English language) and investigating the economic, clinical (therapeutic and medication safety), and humanistic impact (health-related quality of life) of pharmacists’ services, from both hospital and community settings, were included.

**Results:**

The electronic search yielded 430 studies, of which 20 relevant ones were included in this review. Most studies were conducted in India (9/20), followed by Pakistan (6/20), Nepal (4/20) and Sri Lanka (1/20). One study showed a low risk of bias (RoB), 12 studies showed some concern, and seven studies showed a high RoB. Follow-up duration ranged from 2 to 36 months. Therapeutic outcomes such as HbA1c value and blood pressure (systolic blood pressure and diastolic blood pressure) studied in fourteen studies were found to be reduced. Seventeen studies reported humanistic outcomes such as medication adherence, knowledge and health-related quality of life, which were found to be improved. One study reported safety and economic outcomes each. Most interventions delivered by the pharmacists were related to education and counselling of patients including disease monitoring, treatment optimisation, medication adherence, diet, nutrition, and lifestyle.

**Conclusion:**

This systematic review suggests that pharmacists have essential roles in improving patients’ ECHO in South Asian countries via patient education and counselling; however, further rigorous studies with appropriate study design with proper randomisation of intervention and control groups are anticipated.

## Introduction

Pharmacists’ role has shifted from traditional dispensing-focused to pharmaceutical and clinical care services. The professional roles of pharmacists are continuously evolving and focus on helping patients achieve their optimal health outcomes. Pharmacists in many High-Income Countries (HICs) actively participate in multidisciplinary healthcare teams to deliver regular clinical pharmacy service that includes medication reconciliation and review, pharmacotherapy consultation, therapeutic drug monitoring, adverse drug reactions reporting, discharge counselling and solving other medication therapy-related problems [1, 2]. In contrast, the range of pharmacy services is limited and is not up to the standards in low- and middle-income countries (LMICs) as HICs [3]. However, pharmacists in LMICs have been recently reported to participate in ward rounds with other healthcare providers to document and evaluate patients' clinical progress and medication-related issues and develop and implement medication therapy management plans [4, 5].

Currently, more than 50% of all medicines are prescribed and dispensed inappropriately, and only 50% of patients take them properly globally [6, 7]. Irrational antimicrobials use, failure to complete the full course of therapy, missed doses, misuse of drugs, reuse of leftovers, use of sub-therapeutic or supra-therapeutic doses of drugs all promote the emergence of resistance, augmented therapeutic costs and even lead to the patients’ death [6, 7]. Pharmacists in LMICs have the potential to play a pivotal role in promoting rational use of medicines, regulating medication concordance, preventing and resolving drug therapy-related problems, providing drug information and improving pharmacotherapy and health-related quality of life (HRQOL) of patients [8–11]. A systematic review of the impact of pharmacist interventions on patient outcomes, health service utilisation, and costs in LMICs found that pharmacist-delivered services may improve clinical outcomes among patients with diabetes, hypertension, hyperlipidemia, and asthma may improve their HRQOL [3]. Another systematic review that studied the 54 randomised control trials examined the impact of pharmaceutical care using patient outcomes and found that pharmaceutical care effectively improves patient short-term outcomes for several conditions, including diabetes and cardiovascular conditions [12].

Pharmacists can help physicians in selecting the appropriate medication for prescribing. Furthermore, pharmacists can contribute to understanding and reviewing patients’ adherence to prescribed medications, their dosage, and appropriate administration, which will help physicians understand the progress of medication treatment [4, 13–16]. Also, they can contribute to public health promotion via community pharmacies. For instance, tobacco control and cessation services, nutrition and healthy lifestyle management, routine immunisation, infection prevention and control, management of mental health and chronic disease care, and health and environment-related other concerns [4]. Clinical pharmacy practice is in the infant stage in South Asian countries [13, 17]. Although hospital pharmacists are expected to provide clinical pharmacy services, roles are mainly limited to dispensing and material management; on the other hand, pharmacists are reported to have education, skills and confidence in delivering clinical pharmacy services [13]. Clinical pharmacists require skills in clinical practice, critical thinking, therapeutic decision-making, and inter-professional collaborations. Experiential learning and training are essential to gain these skills [13, 17]. Clinical pharmacy services help pharmacists be more patient-focused than traditional dispensing services and gain recognition from policymakers and patients.

Postgraduate educations are key to furthering pharmacists' skills and education. In recent years, such postgraduate programmes have been established in South Asia. For example, clinical pharmacy and pharmaceutical care-related 2-year postgraduate courses were started in Nepal at Kathmandu University (from 2000), Pokhara University and Purbanchal University in 2011 and 2016, respectively, and CIST College, a private college affiliated with Pokhara University in 2017 [18]. Also, Kathmandu University commenced offering a 3-year post-baccalaureate Doctor of Pharmacy (PharmD) course, with two academic years of study plus a year internship in hospital speciality units for a period of 5 years (2010–2015). However, it resumed the earlier 2-year pharmaceutical care programme after 2015 [13, 18]. Similarly, a postgraduate clinical pharmacy course was initiated in India at JSS College of Pharmacy in 1996. Later, a 6-year PharmD course, with five academic years of study and a year of internship in speciality units, was also initiated in 2008, and a 5-year PharmD course was started in Pakistan in 2005 [17].

The South Asian Association of Regional Cooperation (SAARC), a regional inter-government consortium of eight South Asian countries, namely Afghanistan, Bangladesh, Bhutan, India, Maldives, Pakistan, Nepal, and Sri Lanka, serves as an abode of about 26.3% (i.e., 1,940,369,612) of the world population and ranks the first in the whole Asian region [19]. The increased health-related problems and the lack of healthcare resources exponentially with the population surge, health professionals, including pharmacists, have crucial roles in promoting better health-related outcomes. However, even though the role of pharmacists has been well known, and various efforts have been made to establish clinical pharmacy programmes across South Asian countries, less number of pharmacy professionals get chances to actually demonstrate their roles to contribute toward the economic, clinical and humanistic outcomes. Also, limited studies have evaluated the impact of pharmacists’ services on economic, clinical, and humanistic outcomes in South Asian countries. The present review aims to explore the existing evidence so far and assess and evaluate the impact of pharmacists’ services on economic, clinical and humanistic outcomes of patients in South Asian countries.

## Methods

### Study design

This systematic review evaluated the pharmacist's impact on patients’ economic, clinical, and humanistic outcomes in South Asia. It was conducted in accordance with guidelines in the Cochrane Handbook for Systematic Reviews of Interventions [20, 21] and reported according to the Preferred Reporting Items for Systematic Reviews and Meta-Analyses (PRISMA) [22]. The review protocol was registered in the Prospective Register of Systematic Reviews (PROSPERO), with the registration number CRD42021273684.

### Search strategy, selection criteria, data sources and extraction

The **P**opulation, **I**ntervention, **C**omparison, **O**utcome (PICO) elements were used to formulate the research question, eligibility criteria and search strategy, where (P) patients or caregivers who received pharmacists' services; (I) pharmacists' services; (C) patients who did not receive pharmacists' services; and (O) economic, clinical, and humanistic outcomes (ECHO) achieved after pharmacists' services.

The process of identifying studies was performed by (SS and AA). Five databases were searched and reviewed, including PubMed/Medline, Scopus, EMBASE, CINAHL, and Cochrane Library. A manual search of the reference lists of related systematic reviews, all included studies, and all additional relevant reviews was identified in the electronic search. In addition, it was checked to find further research related to this review. All references found as potentially related were conferred with a review team and deduplicated in contradiction to records already retrieved through the electronic searches.

We searched PubMed/Medline, Scopus, EMBASE, CINAHL, and Cochrane Library for relevant articles published from inception to 20th September 2021. Three reviewers (SS, AA and APK) independently participated in the studies' screening and selection processes; they first reviewed relevant titles and abstracts and then relevant full texts based on the eligibility criteria. A fourth reviewer (BS) settled any discrepancy in the same. Original studies [only randomised controlled trials (RCTs)] conducted in the South Asian countries (published only in the English language) and investigating the economic, clinical (therapeutic and drug safety), and humanistic impact of pharmacists, from both hospital and community settings, were included and extracted into Microsoft Excel spreadsheet. Data extraction forms were pilot tested on five studies and revised as needed. The following data were extracted: primary author, publication year, country, study design, setting, sample size, duration, follow-up, characteristics of the study population (mean age and disease states), baseline characteristics of the intervention, comparison groups, the intervention (i.e., pharmacists’ services), outcomes (economic, clinical, and humanistic), and limitations or bias described in the studies. Initially, SS and RS independently extracted the data, which were reviewed by three reviewers (AA, CM and APK). The corresponding authors were contacted by email if data were not reported and/or clarity of the extracted data was required. A consensus among the reviewers resolved any divergence in extracted data. In addition, two reviewers (PT and RS) independently assessed the ROB in studies resolving differences through consensus. All the studies included were synthesised descriptively by following the PRISMA guidelines.

### Eligibility criteria

Original studies (only RCTs) conducted in the South Asian countries (published only in the English language from inception to 20th September 2021) and investigating either the economic (direct medical and non-medical healthcare cost), clinical (therapeutic and drug safety), or humanistic (such as quality of life, medication adherence, knowledge, attitude, practice, patient's satisfaction) impact of pharmacists, from both hospital and community settings, were included in this systematic review. However, we excluded conference abstracts, case reports, conference papers, editorial, opinion papers, reviews, systematic reviews, and study protocols.

### Definitions of health outcomes followed in this systematic review

We followed the ECHO model of the classification of health outcomes put forth by Kozma et al. [23]. We further considered the ECHO model as that clinical outcome (comparative clinical effectiveness research, improved disease or symptom control, safety and/or adverse effect of pharmacotherapy received), humanistic outcomes (patient satisfaction, medication adherence, and patients' HRQOL) and economic outcomes (pharmacoeconomics, reduction in Health Care Costs (HCCs) or utilisation, such as hospitalisations, emergency and/or clinic visits, and/or avoided drug costs) [24–26].

### Nature of intervention

Pharmacists’ professional care includes counselling patients on rational medication use, monitoring medication adherence, monitoring drug interaction, and monitoring beneficial and adverse medication effects. The intervention group (pharmacist-led professional pharmaceutical care or intervention) was compared vs. the control group (usual pharmacy service or medical care or non-pharmaceutical or non-clinical pharmacist care).

### Risk of bias assessment

The randomised studies were assessed using the Cochrane Risk of Bias tool (RoB) [27, 28] independently by two authors. The ROB was categorised as ‘low’, ‘some concern’, and ‘high’ based on essential domains. The ROB was transferred to the computer-based RevMan V.5.3 to generate the ROB graph and summary. Any disagreements on judgment were resolved through the conversation between the authors. Cohen’s kappa index (κ) was used to evaluate the level of agreement between two reviewers in the study selection process, adopting a 95% confidence interval. The agreement between reviewers was based on the following established criteria: κ < 0.20 poor, κ: 0.21–0.40 fair, κ: 0.41–0.60 moderate, κ: 0.61–0.80 good and κ 0.81–1.00 very good agreement [29].

#### Data synthesis

Given the lack of homogeneity of study aims, participants and outcome measures, a narrative approach to data synthesis was undertaken, using text and tables aligned to each of the review objectives.

#### Data analysis

Due to differences in terms of intervention contents, duration, follow-ups, study designs, outcomes measuring instruments, participant demographics, types of interventions delivered by the pharmacist, and settings, data were synthesised narratively, and meta-analysis could not be performed.

## Results

### Study selection

**Fig. 1:**
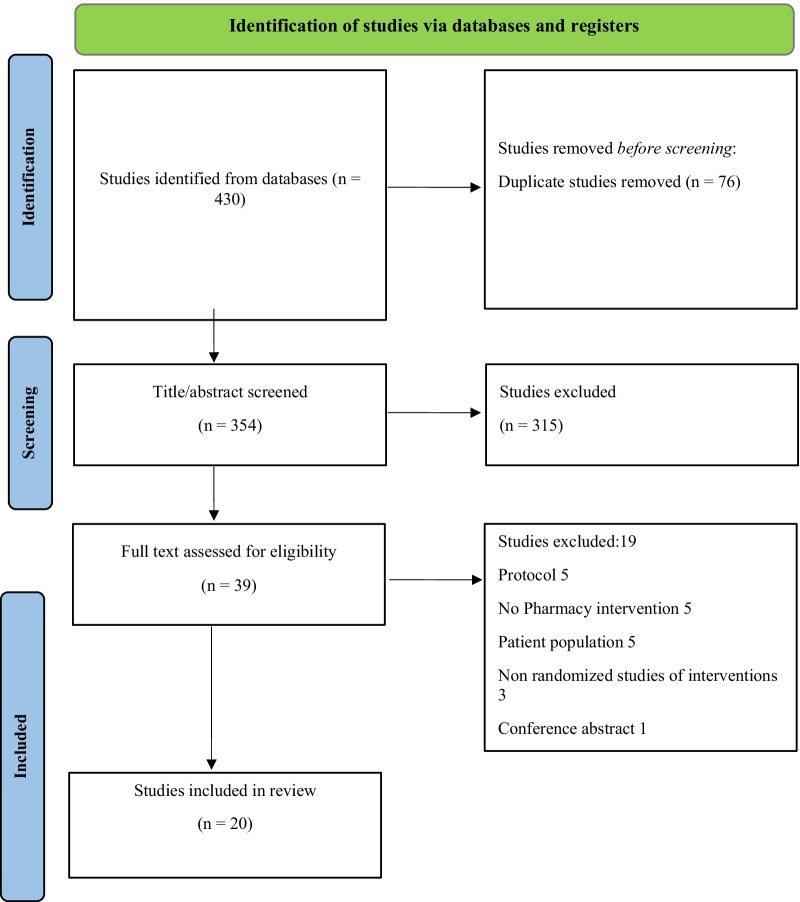
The PRISMA flowchart for included studies

The electronic search yielded a total of 430 studies. After removing duplicates, a total of 354 titles and abstracts were screened against the eligibility criteria. Subsequently, 39 full articles were screened, of which 20 were included in this review (Fig. 1).

### Characteristics of included studies

All the included studies were conducted between 2004 and 2020. The sample size included in 20 studies was 4,357 in total. People living with diabetes, hypertension, depression, asthmatic, human immunodeficiency virus and hepatitis C infection were included in the studies. Most studies were conducted in India (9/20) [30–38] followed by Pakistan (6/20) [39–44], Nepal (4/20) [45–48] and Sri Lanka (1/20) [49].

Variation was found in the pharmacists' provided intervention in South Asian regions. Interventions were based on education, counselling, individualised patient care, pharmaceutical care, and interviews. Nine of the studies were conducted in the outpatient departments [34, 35, 37–39, 41–43, 47], nine were hospital-based [30–32, 39, 40, 44, 46, 48, 49], one in primary care setting [43] and one of the studies was conducted in community pharmacy-based service [33]. In terms of the measured outcomes, included studies reported various outcomes and the follow-up study ranged from 2 to 36 months. Therapeutic outcomes were studied in fourteen studies [30, 32–35, 37–43, 47, 49]. Seventeen studies reported humanistic outcomes [30–42, 44, 45, 47, 48], and one study reported safety and economic outcomes [41, 46]. The frequency of pharmacist intervention sessions was about 15 min for the first session, with follow-up sessions ranging from 10 to 30 min (Table 1).

**Table 1:** Detailed characteristics of studies included in the review

Author (year, country)	Objective	Study design	Sample size (ITT)	Mean age years	Follow-up	Setting	Patients’ description	Control group (CG)	Intervention group (IG)	Outcome		
										Clin	Hum	Eco
Saeed et al. 2021 Pakistan	To determine the impact of pharmacist-led pharmaceutical care on patients’ medication therapy by comparing Patient-Reported Outcomes Measure of Pharmaceutical Therapy for Quality of Life (PROMPT-QOL) between patients on pharmaceutical care (PC) and usual care (UC) models	RCT	*N* = 300 IG = 150 CG = 150	NA	6 months	Tertiary care hospital, Rawalpindi, Pakistan	Inclu: OPD patients aged 18 or over, willing to participate in the study and to take at least three medicines and more Exlcu: Patients with any cognitive impairment and refusal to participate	Participants receiving usual standard care of hospital without pharmacists’ intervention	Participants receiving pharmacists’ counselling and education related to drug use, treatment outcomes, adverse events. etc	×	✓	×
Marasine et al. 2020 Nepal	To evaluate the impact of pharmaceutical service intervention on medication adherence and patient-reported outcomes among patients diagnosed with depression in a private psychiatric hospital in Nepal	RCT	*N* = 212 IG = 107 CG = 105	18 to 65	6 months, two follow-up in interval of 2 months	Out-patient department of B.G. Hospital, Nepal	Inclu: 18–65 years patients with depression, taking at least one antidepressant medication for at least two months. Exclu: Pregnant or lactating mothers with history of psychotic, bipolar disorder or drug abuse, those with cognitive impairment and unable to communicate	Patients receiving usual care as provided in the hospital in regular visits, including usual pharmaceutical service from a pharmacist	A clinical pharmacist delivered face-to-face counselling about depression and associated risk factors for approximately 15 min, educated patients on the use of antidepressant medications, their potential adverse effects, and essential lifestyle modifications to be practised by the patients, and also provided a leaflet of the session	×	✓	×
Chatha et al. 2020 Pakistan	To investigate pharmacist-led interventions to improve adherence to ART for PLHA	RCT	*N* = 66 IG = 33 CG = 33	IG = 36. 18 ± 12.24 CG = 31.39 ± 9.53	2 months, two follow-ups of 30 min duration	Pakistan Institute of Medical Sciences (PIMS)	Inclu: HIV positive, > 18 age, taking ART for > 3 months Exclu: Patients having preliminary baseline blood tests, pregnancy, or cognitive impairments	Participants received a single education and counselling session when their physician-led ART was started	Pharmacist-provided counselling was tailored to each social factor focused on personal barriers to taking medication and was aimed at helping participants understand their medication-taking behaviours while acknowledging the actions needed to maintain a high-level of adherence. It also included advice on the potential negative impact of diet and supplementary herbs or medicines on the effectiveness of ART	✓	✓	×
Ali et al. 2019 Pakistan	To evaluate the impact of clinical pharmacy interventions on treatment outcomes, HRQOL, and medication adherence among hepatitis C patients	RCT	*N* = 931 IG = 465 CG = 466	42.35 ± 1.9	3 months, three follow-up visits	Gastroenterology OPD of SIMS, Lahore and PIMS, Islamabad, Pakistan	Inclu: Patients confirmed with HCV-positive and aged ≥ 18 years who presented to the GED and who started direct acting antiviral (DAA) treatment Exclu: Pregnants or patients co-infected with HBV, HDV or autoimmune hepatitis	They receive usual care from hospital staff. However, they did not receive pharmacists’ intervention such as counselling sessions	Clinical pharmacists provided individualised patient care, including direct patient monitoring, education on life-style modifications, and counselling on the appropriate use of HCV medication. Clinical pharmacy services were continued until treatment was completed	✓	✓	×
Javaid et al. 2019 Pakistan	To demonstrate the pharmacist-led improvements in glycaemic, blood pressure and lipid controls in T2DMpatients	RCT	*N* = 244 IG = 123 CG = 121	50 ± 9.2	9 months with 3 follow-ups; 15–30 min	Primary care facility, Murad clinic Lahore, Pakistan	Inclu: Uncontrolled T2DM patients (HbA1c > 8%), age > 18 years, Hb > 13 mg/dL with or without concomitant disease Exclu: Those with cognitive impairment, below 18 years of age and missing visits in the past six months	Patients in the CG continued treatment from physicians, and nurses provided regular check-ups	Pharmacist performed PWDT, CORE, PRIME. PRIME include interaction, mismatch, non-adherence, ADRs, monitoring and screening of patients at each follow-up, 15 to 30 min average interaction time	✓	✓	×
Yadav et al. 2019 Nepal	To evaluate the effect of a pharmacists’ interventions on asthma control, HRQOL and inhaler technique in adult patients suffering from asthma	Pre- and post- interventional study	*N* = 72 IG = 36 CG = 36		4 months,	OPD of Crimson Hospital, Rupandehi, Nepal	Inclu: Patients aged ≥ 18 years, clinically diagnosed with asthma with or without co-morbidities and who were on inhalers and/or medications for their asthma Exclu: Asthmatic patients admitted in ED	No intervention was made till the completion of the study	The intervention was carried out outside the Medicine-OPD, and patients were counselled for nearly 20–25 min. Patients were later provided counseled with leaflets. The video-aided materials were shown to the patients at their follow-ups	✓	✓	×
Gorutla et al. 2019 India	To evaluate the impact of pharmacist- delivered counselling on KAP levels and control of BP among hypertensive patients from various regions of Anantapur district	Prospective open-labelled, RCT	*N* = 102 IG = 95 CG = 97	IG: 43.7 ± 9.11; CG: 43.9 ± 8.26	6 months, 2 follow-ups	Medicine OPD of NGO hospital in Anantapur district, Andhra Pradesh, India	Inclu: Hypertensive patients with ≥ 18 years of age with co-morbidities and who could respond in English/Tamil version of questionnaires. Exclu: Those who were refused to participate and respond to Telugu/English version of questionnaires	The participants in the CG followed the usual care given by the physicians	Pharmacists provided face-to-face counselling on hypertension, regular monitoring of BP and body weight, DASH diet, physical exercise, stress management, salt restriction, lifestyle changes (smoking and alcohol), and regular intake of medications as per the physicians’ instruction to the IG patients	✓		
Abdulsalim et al. 2018 India	To evaluate the effectiveness of a structured pharmacist-led intervention programme on medication adherence among COPD patients in India	Open labelled RCT	*N* = 260 IG = 130 CG = 130	IG = 60.60 ± 7.9 CG = 61.1 ± 8.4	3 years, four times follow-up	Kasturba Medical College Hospital, Manipal, India	Inclu: Confirmed diagnosis of COPD as per GOLD guideline	CG received standard hospital care, but did not receive intervention provided by the clinical pharmacist	Clinical pharmacist counselled patients for 15–20 min and provided information on (1) importance of medication adherence, (2) dose and frequency of medications, (3) need for smoking cessation, (4) simple exercise, (5) proper use of inhaler devices and (6) need for timely monitoring of medicines using PILs	✓		
Cooray et al. 2018 Sri Lanka	To examine the impact of a culturally appropriate health-education on lifestyle modification and self-management of patients with diabetes to improve their glycaemic control and delay disease complications	Descriptive, cross-sectional and randomised intervention study	*N* = 166 IG = 110 CG = 56	56.2 ± 8.95	12 months, two follow-up	Two main tertiary care facilities in western and south- ern provinces of Sri Lanka	Inclu: T2DM patients aged > 18 years Exclu: Pregnant, GDM, T1DM, patients on haemodialysis, who were unable to speak or understand Sinhala	CG patients received usual care and the same questionnaires as the IG patients but did not receive health education sessions	Structured health education programme covering pathogenesis, progression and complications of T2DM; importance of proper management and follow-ups, and demonstration on blood glucose monitors and insulin pens were given. Also, education on medications’ mode of action, side effects, and adherence to medication on disease prognosis and development of complications	✓	×	×
Amer et al. 2018 Pakistan	To evaluate the effect of pharmacists’ educational intervention to patients with hypertension to improve their knowledge, adherence to medicines, blood pressure control and HRQOL	RCT	*N* = 384 IG = 192 CG = 192	NA	9 months with 3 follow-ups	Polyclinic hospital of Islamabad	Inclu: Hypertension out-patients who could speak or write Urdu, who visited cardiology section of the hospital and who were aged > 30 years, and who were taking antihypertensive medications for the last 6 months. Exclu: Pregnant women, those with co-morbidities, having dementia, immigrants and those aged < 30 years and > 70 years	No educational sessions were provided, but only standard care (provided by the physicians during scheduled visits to the hospital) was provided	Pharmacists conducted interviews of patients at each visit, identified causes of non-adherence to medications, and provided disease-related education to the patients (lifestyle education, medication counselling to increase their knowledge about hypertension, adherence to medications, and HRQOL). A printed booklet (in Urdu language) of HTN-related educational material was also provided to the patients	✓	✓	×
Upadhyay et al. 2016 Nepal	To report the impact of pharmacist-supervised intervention through pharmaceutical care programme on DHCs among the newly diagnosed diabetics in Nepal	RCT	*N* = 162 IG = 108 CG = 54	49.14 ± 12.56	12 months, 4 times follow-up	Manipal teaching hospital, Pokhara, Nepal	Inclu: Newly diagnosed T1DM and T2DM patients with age 16 years and above Exclu: Pregnant and mentally incompetent	Usual care without specific care by pharmacist	Education and counselling about different aspects of DM and its management and the correct use of antidiabetic medications were given to the test group by the pharmacist	×	×	✓
Upadhyaya et al. 2015 Nepal	To determine the baseline satisfaction level of newly diagnosed diabetics and explore the impact of pharmaceutical care intervention on patients’ satisfaction during their follow-ups in a tertiary care teaching hospital in Nepal	Interventional pre-post non-clinical RCT	*N* = 152 IG = 102 CG = 50	49.14 + 12.56	18 months, four times follow-up	Manipal teaching hospital, Pokhara, Nepal	Inclu: T1DM and T2DM patients aged 16 years and above. Exclu: Pregnant women and mentally incompetent patients	CG patients did not receive pharmaceutical care intervention from pharmacist and maintained on usual care obtained from physician/nurses throughout the study	IG received information about diabetes such as its types, sign and symptoms, reasons for high BG, risk factors, acute and chronic complications and role of pharmacological (anti-diabetic medications) and non-pharmacological (lifestyle modification, diet and exercise) measures in managing DM and administration of insulin at home	✓		
Saleem et al. 2013 Pakistan	To assess the impact of an educational intervention provided to hypertensive patients through hospital pharmacists to improve their knowledge on HTN, their adherence to the medications and HRQOL	RCT	*N* = 385 IG = 193 CG = 192	39 ± 6.5	Nine months, 3 follow-up visits; first visit 15 min, later visits of 10 min	Cardiac units of SPH and BMCH located in Quetta	Inclu: Out-patients aged 18 or over with an established medical diagnosis of HTN, familiarity with Urdu, and who were taking antihypertensive medication for the last 6 months. Exclu: Patients with dementia, pregnancy and immigrants	The control group had no hospital pharmacists' involvement, and only received traditional service provided by the hospitals (receiving prescription orders, counselling about medication use and information about follow-up visits)	Hospital pharmacist-provided health education about HTN (nature, management, treatment and recommended diet and lifestyle modification), medication adherence and its importance in pharmacotherapy and HRQOL (conceptualisation and importance in treatment outcomes for hypertensive patients). The pharmacist also provided a pocket-sized educational booklet about HTN, information leaflets and medication adherence cards (all in Urdu) during the counselling process	✓	✓	×
Wal et al. 2013 India	To assess the effects of pharmaceutical care interventions in patients with essential HTN	RCT	*N* = 142 IG = 72 CG = 70	IG = 59.50 ± 8.55 CG = 60.62 ± 8.32	4 months	Medicine OPD at Lakshmi Pat Singhania, Institute of Cardiology, Kanpur	Inclu: Newly diagnosed hypertensives aged 20 to 75 years and who had an average DBP > 90 mmHg or an average SBP > 140 mmHg and who were with or without other co‑morbidities. Exclu: Those who refused to come on the scheduled follow-ups	CG did not receive any pharmaceutical care	Patients were counselled on their antihypertensive medications, indications of medicine, specific instructions on the administration of medication, adverse effects, drug interactions, and the importance of adherence to diet and medication therapy using health education materials (in Hindi and English). Appropriate storage conditions of medications, mean obtaining follow-up supplies of medication, and action to be taken in the event of a missed dose were made clear to the patients.	✓	×	×
Ramanath et al. 2012 India	To know the impact of clinical pharmacists’ interventions on medication adherence and HRQOL	RCT	*N* = 52 IG = 26 CG = 26	NA	7 months, two follow-*up	Medicine IPD or OPD at Adichunchanagiri Hospital and Research Center, India	Inclu: Patients aged 18 years or above and who were taking medication for HTN for over 6 months Exclu: Patients with more than four co-morbidities	CG did not provide any counselling and PILs at the baseline and first follow-up. However, they were provided with oral instruction and PILs at the end of the second follow-up	IG patients were counselled about medications, lifestyle changes, and disease management and informed if any unintended effects of medications occurred at any follow-ups.	✓	✓	×
Malathy et al. 2011 India	To assess the baseline levels of KAP of diabetics, develop a counselling programme, and assess whether this intervention could produce any improvement in DM awareness and practices	RCT	*N* = 207 IG = 137 CG = 70	IG = 52.07 ± 9.47 CG = 51.02 ± 9.83	9 months	Two selected multispeciality hospitals and one diabetic clinic in Erode, Tamilnadu, India	Inclu: DM diagnosed among patients aged > 30 years Exclu: Pregnant women and paediatric patients	Usual care without counselling but the group received pharmacists’ counselling at the end of the study only	Pharmacist counselled patients in their local language for 20–25 min on each visit at 1-month intervals over 3 months Pharmacist explained pathophysiology and etiology of DM, acute and chronic complications, importance of BG control lifestyle changes (e.g., exercise, smoking cessation, etc.), nutrition and foot care. After the first counselling session, the test group patients were provided with printed handouts in their local language (Tamil) containing information on DM and dietary and lifestyle changes.	✓	✓	×
Sriram et al. 2011 India	To evaluate the impact of pharmaceutical care on HRQOL among T2DM patients	Prospective, CG versus IG clinical trial	*N* = 120 IG = 60 CG = 60	IG = 53.65 ± 2.38 CG = 57.98 ± 2.62	8 months	Medicine department of, multi-speciality tertiary care teaching hospital, Coimbatore, India	Inclu: T2DM patients aged > 18 years. Exclu: Pregnant women, mentally incompetent patients and critically ill patients	CG patients did not receive any pharmaceutical care	IG received pharmaceutical care, such as medication counselling, instructions on dietary regulation, exercise and other lifestyle modifications using PILs, diabetic diet chart (in both Tamil and English) and diabetic diary.	✓	✓	×
Adepu et al., 2010 India	To assess the influence of structured patient education on therapeutic outcomes among patients with T2DM and HTN	Prospective randomised and interventional study	240	57	3 months	Medicine OPD of a South Indian tertiary care teaching hospital, India	Inclu: T2DM or hypertensive patients who knew Kannada or English language Exclu: Pregnant women with GDM or pre-eclampsia, those with uncontrolled and complicated DM and HTN, or those who had any significant cardiac complications in the last six months	CG patients received detailed education only at the final follow-up visit	IG patients received education regarding the disease, medication, diet and lifestyle modification at baseline and on each follow-up.	✓	✓	
Adepu et al. 2007 India	To assess the impact of pharmacist-provided counselling on treatment outcomes and HRQOL among T2DM patients by improving their KAP	Randomised prospective controlled study	*N* = 70 IG = 35 CG = 35	IG = 51.45 ± 12.27 CG = 53.77 ± 10.35	6 months	Two selected community pharmacies in Calicut, Kerala, India	Inclu: T2DM patients aged > 30 years who were of either gender and were treated with either diet alone or diet and OHAs. Exclu: Paediatric patients, pregnant and those with uncontrolled DM with complication	Usual care without counselling but received pharmacists’ counselling and PILs at the end of study only	IG received counselling on their disease, drugs, diet and lifestyle modification, and PILs highlighting the disease, diet, and lifestyle modifications.	✓	✓	×
Ponnusankar et al. 2004 India	To assess the impact of medication counselling on patients’ medication knowledge and improvements in their adherence	Randomised interventional study	*N* = 90 IG = 30 CG = 60	41 to 60	9 months, two follow-up	Out-patient clinic of private hospital, India	Inclu: Patients with chronic conditions (HTN, DM, CV conditions, and bronchial asthma) since at least 6 months. Exclu: Patients with cognitive or perceptual problems	The usual care group did not receive any counselling	Counselled group received medication counselling from the pharmacist for 15–20 min	×	✓	×

*ADRs* adverse drug reactions, *AIDS* acquired immunodeficiency syndrome, *ART* antiretroviral therapy, *BG* blood glucose, *BP* blood pressure, *Clin* clinical outcomes, *COPD* chronic obstructive pulmonary disease, *DASH* dietary approach to stop hypertension, *DBP* diastolic blood pressure, *DM* diabetes mellitus, *ED* emergency department, *Exclu* exclusion criteria, *GDM* gestational diabetes mellitus, *GED* gastroenterology department, *GOLD* global initiative for chronic obstructive lung disease, *Hb* haemoglobin, *HBV* hepatitis B virus, *HCV* hepatitis C virus, *HDV* hepatitis D virus, *HIV* human immunodeficiency virus, *HTN* hypertension, *Inclu* inclusion criteria, *IPD* inpatients department, *NGO* non-governmental organisation, *OHAs* oral hypoglycaemic agents, *OPD* outpatients department, *SBP* systolic blood pressure, *PILs* patient information leaflets, *RCT* randomised controlled trial, *T1DM* type 1 diabetes mellitus, *T2DM* type 2 diabetes mellitus, *PLHA* people living with HIV and AIDS, *Thera* therapeutics, *Hum* humanistic outcomes, *Eco* economic outcomes, *FIVs.* follow on interventions, *DHQ* district headquarter hospital, *BMCH* Bolan Medical Complex Hospital, *BMI* body mass index, *MDR-TB* multidrug-resistant tuberculosis, *RIVs.* rejected interventions, *HRQOL* health-related Quality of Life, *SPH* Sandeman Provincial Hospital, *SIMS* Services, Institute of Medical Sciences, *PIMS* Pakistan Institute of Medical Sciences, *PWDT* pharmacist’s work-up of drug therapy, *CORE* condition, outcome, regime, evaluation, *PRIME* Problem, Risk, Interaction, Mismatch, Efficacy, *CKD* chronic kidney disease, *KDOQI* Kidney Disease Outcomes Quality Initiative, *GFR* glomerular filtration rate, *CG* control group, *IG* intervention group

### Risk of bias

**Fig. 2:**
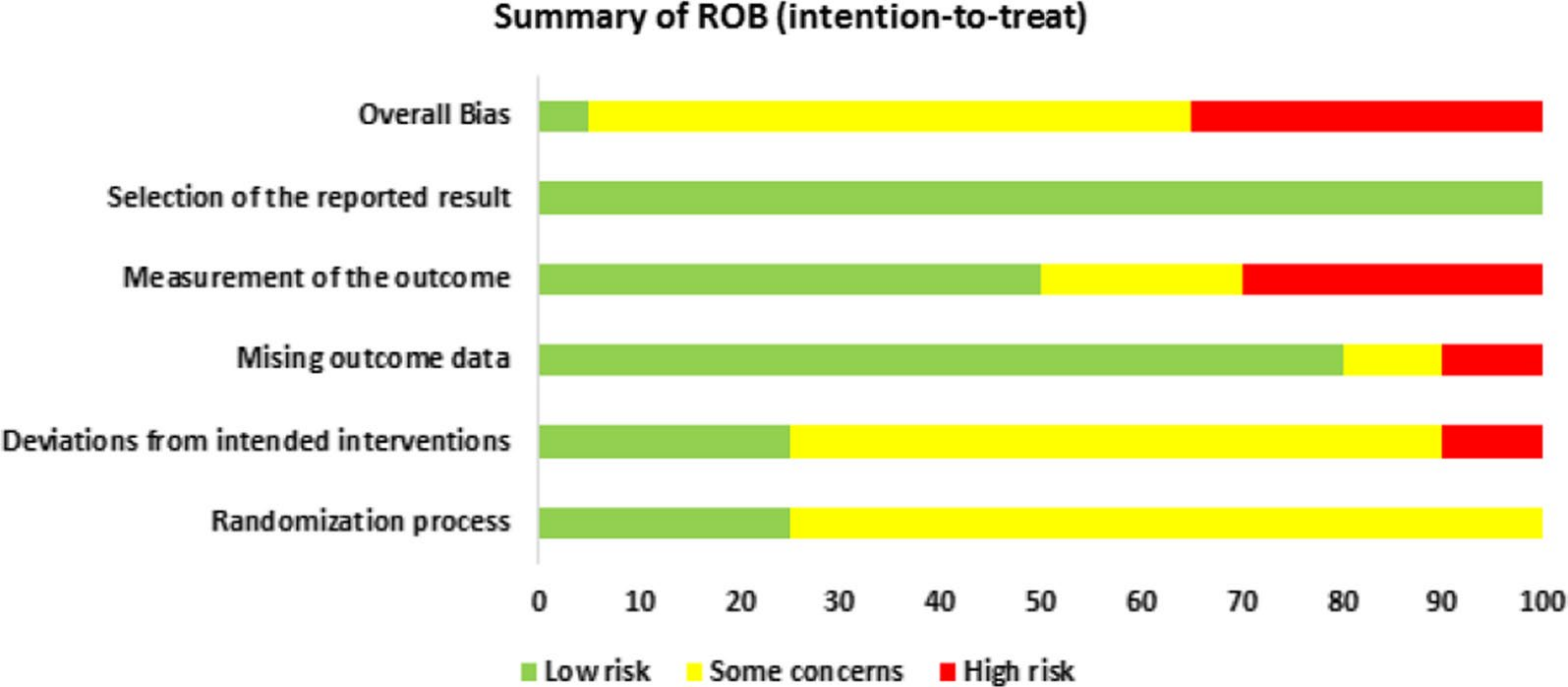
Summary of risk of bias

**Fig. 3:**
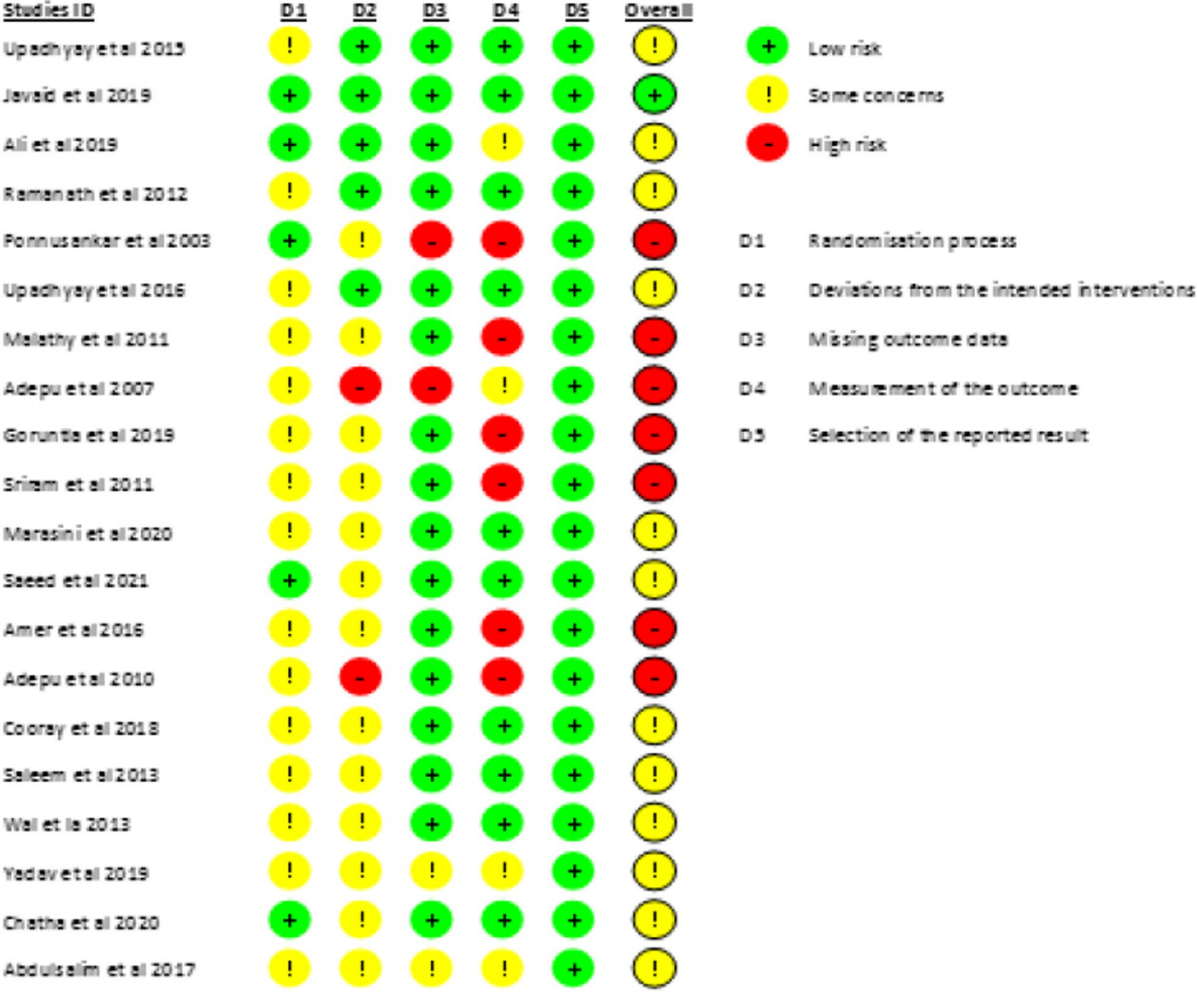
Details of risk of bias of included studies

Overall, the RoB was generally variable across domains. The summaries show that one study showed a low ROB [43], twelve studies [30, 35, 36, 39, 41, 42, 44–49] showed some concern, and seven studies showed a high RoB [31–34, 37, 38, 40]. Figure 2 shows the assessments of each RoB item for each included study. Most of the common causes of bias in the included studies were participation randomisation process, missing outcome data and measurement of outcomes. Only five studies indicated concealment allocation [31, 41–44]. In addition, two studies highlighted the high-risk bias in providing more than one questionnaire for their intervention purpose to collect their various outcomes [33, 38]. Two studies [31, 33] showed a high ROB in reporting data outcomes and being influenced by the output of outcome data. Regarding the measurement of outcomes, six studies have reported a high RoB, probably influenced by the assessor’s knowledge [31, 32, 34, 37, 38, 40]. Notably, all studies reported a low RoB in selecting the findings report (Fig. 3).

### Pharmacist interventions

Most interventions delivered by pharmacists were divided into education and counselling, which were further divided into education and counselling of diseases, advantages of monitoring of disease, management and treatment, medication adherence, diet, nutrition, and lifestyle. In addition, there was the provision of booklets, written materials, leaflets, and written information to aid education and counselling in some studies. Table 1 provides the details of the intervention delivered by the pharmacist in the individual included study. Similarly, Table 2 summarises the interventions delivered by the pharmacist in included studies.

**Table 2:** Interventions delivered by the pharmacist in South Asian countries

	Counselling on					Provision of booklets, written materials, leaflets, and written information	Others
	Diseases	Regular monitoring of disease	Management and treatment	Medication adherence	Diet, nutrition and lifestyle		
Saeed et al. 2021			√				d
Marasine et al. 2020	√		√		√	√	d
Chatha et al. 2020				√	√		c
Ali et al. 2019			√		√		b
Javaid et al. 2019							a, d, e
Yadav et al. 2019						√	f
Gorutla et al. 2019	√	√	√	√	√		g
Abdulsalim et al. 2018	√	√	√	√	√		
Cooray et al. 2018	√	√	√	√			
Amer et al. 2018	√		√	√	√	√	a
Upadhyay et al. 2016	√	√	√		√		
Upadhyaya et al. 2015	√		√	√	√		
Wal et al. 2013	√		√	√			
Saleem et al. 2013	√		√	√	√	√	
Ramanath et al. 2012					√		d
Malathy et al. 2011	√	√	√		√	√	
Sriram et al. 2011	√		√		√	√	
Adepu et al. 2010	√		√		√		
Adepu et al. 2007	√		√		√	√	
Ponnusankar et al. 2004			√				

^a^Patient interview and obtaining medical history; ^b^direct patient monitoring; ^c^education-related supplement drugs and herbs; ^d^unwanted effects of drugs or adverse drug reactions; ^e^drug–drug interaction; ^f^video-aided material; ^g^stress management

### Economic outcomes

Only one study has reported the economic impact of pharmacist care in the South Asian region. Upadhyay et al. reported a significant difference in direct healthcare cost from control group to two intervention groups that are at 6 months (*p* = 0.009, *p* = 0.010, respectively), 9 months (*p* = 0.005, *p* = 0.001, respectively), and 12 months (*p* = 0.001, *p* = 0.001, respectively) [46]. The direct healthcare cost was the total medical and non-medical expenses from the patient's perspective during treatment. The pharmacist-provided intervention significantly decreased the direct healthcare costs of patients in test groups during their follow-ups with a greater reduction in drug costs and investigation costs.

### Clinical outcomes (therapeutic and safety outcomes)

#### Therapeutic outcomes

Six studies showed that pharmacists' interventions significantly reduced systolic blood pressure (SBP) and diastolic blood pressure (DBP) in hypertension patients compared to the control group (CG) [35, 37–40, 43]. Amer et al. in their study where pharmacist-provided pharmaceutical care to hypertensive patients, reported that pharmacist-led intervention significantly improved hypertension with SBP (131.81 ± 10.98 mmHg) and DBP (83.75 ± 6.21 mmHg) among the patients of the intervention group (IG) [40]. Javaid et al. found that individuals in the intervention arm improved their SBP (mean difference = IG: 21.1 vs. CG: + 6.1; *p* 0.001) and DBP (mean difference = IG: 7 vs. CG: + 4; *p* 0.001) more than those in the control arm [43].

Three studies indicated improvement in pre-existing diabetic conditions with a reduction in their HbA1c values [34, 43, 49]. Sriram et al. reported that the average HbA1c values decreased from 8.44 ± 0.29% to 6.73 ± 0.21% (*p* < 0.01) IG [34]. Similarly, Javaid et al. mentioned that the intervention group exhibited significant improvement in HbA1c outcome in both pre/post groups and control vs. intervention groups. (10.3 ± 1.3 vs. 9.7 ± 1.3, *p* < 0.001, I; 10.9 ± 1.7 vs. 7.7 ± 0.9, *p* < 0.0001) [43]. However, Yadav et al. showed improvement in asthmatic conditions where a change in the mean score of asthma control in the test group (*p* = 0.001) was reported, which was more significant than the control group (*p* = 0.099) [47]. Two studies reported improving certain lipid profile components [32, 49]. Malathy et al. showed triglycerides levels in the test group decreased considerably from 150.9 mg/dL to 140.6 mg/dL (*p* < 0.001) as compared to the control group(155.7 mg/dL to 148.5 mg/dL) [32]. In the test group, high density lipoprotein levels increased considerably from 34.9 mg/dL to 36.6 mg/dL (*p* = 0.05) [32]. Cooray et al. reported a reduction in body mass index readings, with the intervention group exhibiting 24.4 kg/m^2^ compared to the control group 24.9 kg/m^2^ after 6 months of intervention [49]. Table 3 summarises the pharmacist’s impact on patients’ outcomes regarding therapeutic, humanistic, and safety outcomes.

**Table 3:** Summary of the pharmacists’ impact on patients’ economic, clinical and humanistic outcomes

Authors	Economic		Therapeutic		Safety		Humanistic	
	IG vs. CG	BI vs. AI	IG vs. CG	BI vs. AI	IG vs. CG	BI vs. AI	IG vs. CG	BI vs. AI
Saeed et al. 2021	–	–	–	–	–	–	QoL^++^	–
Marasini et al. 2020	–	–	–		–		Adherence^++^ QoL*	Adherence^#^ QoL^#^
Chatha et al. 2020	–		CD4 cell count^+^	CD4 cell count^+^	–		Adherence^+^	Medication belief^#^ Adherence^#^
Ali et al. 2019	–	–	SVR 12^++^ ETR*	–	ADE^#^ DDI^#^	–	Adherence^++^ QoL*	QoL^++^
Javaid et al. 2019	–	–	HbA1C^++^ eABG^+^ SBP^++^ DBP^++^ Cholesterol^++^ TG^++^ HDL* LDL-C^++^ VLDL-C^++^ Serum creatinine^++^ eGFR^++^	HbA1C^++^ eABG^++^ SBP^++^ DBP^++^ Cholesterol^++^ TG^++^ HDL* LDL-C^++^ VLDL-C^++^ Serum creatinine^++^ eGFR^++^	–	–	–	–
Yadav et al. 2019	–	–	Level of asthma control^++^	Level of asthma control^++^	–	–	QoL^++^	QoL^#^ Knowledge of MDI use^++^
Gorutla et al. 2019	–		SBP^+^ DBP^+^	SBP^#^ DBP^#^	–	–	Knowledge^++^ Attitude^++^ Practice^++^	Knowledge^#^ Attitude^#^ Practice^#^
Saleem et al. 2015	–	–	SBP^++^ DBP^++^	SBP^++^ DBP^++^	–	–	Knowledge^++^ Adherence^++^ QoL^—^	QoL^–^
Amer et al. 2018	–	–	SBP^++^ DBP^++^	SBP^++^ DBP^++^	–	–	Knowledge^++^ Adherence^++^ QoL^++^	Knowledge^++^ Adherence^++^ QoL^++^
Abdulsalim et al. 2018	–		–		–		Adherence^++^	Adherence^#^
Cooray et al. 2018	–	–	HbA1c^#^ SBP^#^ DBP^#^ TC^#^ HDL^#^ TG^#^ LDL* BMI*	HbA1c^++^ TC^++^ LDL^++^ SBP* DBP* BMI^++^	–	–	–	–
Upadhyay et al. 2016	DHCs^++^	DHCs^++^	–	–	–	–	–	–
Upadhyay et al. 2015	–	–	–	–	–	–	Satisfaction score^++^	Satisfaction score^++^
Wal et al. 2013	–		SBP^#^ DBP^#^	SBP^++^ DBP^++^	–	–	QoL^#^	QoL^++^
Ramanath et al. 2012	–	–	BP*	BP^#^	–	–	Adherence^++^ QOL^++^	Adherence^#^ QOL^#^
Malathy et al. 2011	–	–	PPBG^#^ TGL^#^ TC^#^ HDL^#^ LDL^#^ VLDL^#^	PPBG^++^ TGL^++^ TC* HDL^+ ^ LDL* VLDL*	–	–	Knowledge^#^ Attitude^#^ Practice*	Knowledge^++^ Attitude^++^ Practice*
Sriram et al. 2011	–	–	FBG^#^ HbA1c^#^	FBG^++^ HbA1c^++^	–	–	QoL^#^ Treatment satisfaction^#^ BMI^#^	QoL^++^ Treatment satisfaction^++^ BMI^+^
Adepu et al. 2010	–		SBP^#^ DBP^#^ CBG^#^	SBP^+^ DBP^+^ CBG^++^	–		KAP^#^ Adherence^#^	KAP^++^ Adherence^#^
Adepu et al. 2007	–	–	BG^#^	BG^#^	–	–	QoL^#^ KAP^#^	QoL^#^ KAP^#^
Ponnusankar et al. 2004	–		–		–		Medication Compliance^#^ Medication knowledge^#^	Medication knowledge^++^

IG vs. CG intervention group versus control group, BI vs. AI before intervention versus after intervention, *no significant (*p* > 0.05) difference but similar outcome between intervention and control group, ^+^significant (*p* < 0.05) result in favour of intervention group, ^++^significant (*p* < 0.01) result in favour of intervention group, ^#^positive effect in favour of intervention group but no statistical test performed, ^–^significant (*p* < 0.05) effect in favour of control group; ^—^significant (*p* < 0.01) effect in favour of control group; SVR 12 = sustained virological response at 12 weeks, *FBS* fasting blood sugar, *ADE* adverse drug event, *CBC* complete blood count, *RFT* renal function test, *ETR* end-of-response, *BMI* body mass index, *SBP* systolic blood pressure, *DBP* diastolic blood pressure, *eGFR* estimated glomerular filtration rate, *LDL-C* low density lipoprotein-cholesterol, *VLDL-C* very low density lipoprotein-cholesterol, *TGL* triglyceride, *HDL* high density lipoprotein, *BG* blood glucose; *DHCs* direct healthcare costs, *QoL* quality of life, *KAP* knowledge, attitude and practice

#### Safety outcome

According to Ali et al. intervention groups showed positive outcomes based on adverse drug events (8.2%) compared to the control group (10.5%) [41]. However, no statistical test was performed to create an evidence-based analysis finding [41].

#### Humanistic outcomes

Six studies reported improving adherence through pharmacists’ interventions [30, 39–42, 45]. In terms of knowledge, pharmacists’ interventions elevate the knowledge level among patients. The study by Amer et al. was the only study that showed improvement in both groups (intervention vs. control and pre vs. post-study) with an increase in the mean knowledge score about hypertension (18.18 ± 4.00) [40]. Regarding the quality of life, seven studies showed higher score levels [30, 34, 35, 40, 41, 44, 47]. Furthermore, the HRQOL of patients in the intervention group who received pharmaceutical care improved significantly from baseline (*p* 0.0001; compared to the control group (*t* = 6.957), in which the HRQOL was much lower (*p* 0.0001; *t* = 3.273). However, one study, Saleem et al. showed no significant impact on the HRQOL [39]. Apart from that, one study showed significant (*p* < 0.001) improvements in patients' satisfaction scores in the test groups [48].

## Discussion

To our knowledge, this is the first systematic review conducted to include widespread evidence of pharmacists' services provided by the pharmacist in South Asian countries. This systematic review incorporates evidence from 20 studies in which the primary intervention provided by pharmacists/clinical pharmacists was education, counselling and monitoring on management and treatment of diseases.

### Impact of pharmacists’ services on economic outcomes

Pharmacists' services were found to be significant in improving the economic outcomes of patients, which aligns with findings of other studies conducted on HICs [50, 51]. Monte et al. (2009) started a MedSense programme, a pharmacist-led patient-centred pharmacotherapy management programme in the USA and reported that cardiovascular-related costs were decreased by USD 112 at 6 months and by USD 295 at 12 months periods [50]. Wu et al. (2018), in a study conducted at three US Veteran Health Administration hospitals, reported that pharmacist-delivered care yielded a comparable improvement in cardiovascular risk factors from baseline than the conventional pharmacist-minus care, while outpatient care costs decreased among the patients with T2DM. Also, HCCs in the intervention group decreased by USD 795 below baseline levels compared to the continuous increase of USD 501 in the usual care arm [51]. When pharmacists-focused care is provided to the patients, the cost of disease management is somewhat reduced in the long run, and the patients get value for their invested expenses in health.

### Impact of pharmacists’ services on clinical outcomes

This systematic review determined the significant positive impact of pharmacist’s service in improving the clinical outcomes of patients. The evidence on pharmacists’ role in providing clinical services showed that involvement of pharmacists in the disease management process leads to better health outcomes in patients with chronic conditions such as T2DM, and cardiovascular disease. Comparable findings were observed in similar studies conducted in different countries such as Nigeria, Brazil, Singapore, and Egypt [52–54]. David et al. (2021) reported that pharmacist-delivered care significantly improved glycemic control by reducing HbA1c levels in patients with uncontrolled type 2 diabetes mellitus (T2DM) at a tertiary hospital in Nigeria [52]. Clinical pharmacy services such as health education and health literacy empowerment, drug dispensing with counselling, medication reviews, and comprehensive medication management positively impact ECHO in the quasi-experimental before-and-after study conducted in Brazil [53]. Similarly, a systematic review on clinical pharmacy services in chronic kidney disease (CKD) also concluded that the pharmacist interventions led to improvement in creatinine clearance (CrCl), parathyroid hormone (PTH) and calcium levels c in CKD patients [55]. Siaw et al. (2017) presented the indispensable role of the pharmacist as a member of multidisciplinary healthcare professionals to promote better clinical outcomes in chronic disease patients [54].

### Impact of pharmacists’ services on humanistic outcomes

Pharmacist services were significant in improving patients’ humanistic outcomes, which is similar to the findings of a systematic review [56] and some European studies [57, 58]. Clinical pharmacy interventions improved glycemic control and HRQOL, and reduced adverse events (AEs) and costs of T2DM management [56]. RCT performed in community pharmacies in the Netherland documented that clinical medication services over 6 months increased EuroQol Visual Analogue Scale-measured HRQOL level by 3.4 points (from 0.94 to 5.8) among the older patients [58]. The Northern Ireland pharmacist-directed medicines optimisation clinic showed positive cost–benefit effects and patient-centred humanistic outcomes such as beliefs about pharmacotherapy, HRQOL and patient satisfaction with the intervention. These all led to a reduced frequency of emergency department visits and general practitioner consultations even during the post-discharge periods [57].

### Implications for practice and research

The clinical pharmacy services in most South Asian countries are still in the developing phase. As a result, recognition of the clinical roles of pharmacists by other healthcare professionals is still a challenge [59, 60]. However, barriers could be addressed by involving pharmacists in collaborative care, building trust, demonstrating the value of pharmacists in health care teams, and strategically engaging stakeholders, including legal departments, in developing the collaborative practice process. Moreover, initiatives from the professional council at a national level to start clinical residency and certification programmes can be taken in South Asian countries to strengthen pharmacists’ ability to take better responsibility for pharmaceutical care.

Furthermore, pharmacists' continual professional development programmes must be introduced within health facilities to keep them updated with the recent findings on the healthcare systems [13, 61, 62]. Likewise, the benefits and cost-effectiveness of clinical pharmacist interventions should be well studied and implemented to make the pharmacists’ role more recognisable. Appropriately designed studies with standardised outcome measurements, longer duration of pharmacists’ intervention, interventions’ frequency and content are necessary to improve the clinical outcomes [63]. The findings of this review are believed to benefit and make the policymakers in South Asia aware of selecting relevant pharmacist interventions based on the availability of their resources.

### Strengths and limitations

To date, there are numerous reviews from developed and upper-middle-income countries regarding the impact of pharmacist care. However, only one systematic review has been reported from South Asian countries, showing that pharmacists' participation in the healthcare team improves patients’ health outcomes. The findings of the current review align with this fact and suggest that the provision of clinical residency training to pharmacy graduates can play a crucial role in improving patient health outcomes and saving total healthcare costs.

Nevertheless, there are some limitations to this review. Firstly, only peer-reviewed published studies were included in this review to avoid bias, and the unpublished ones were excluded which could provide further details. Secondly, only one or a maximum of two studies were found for each outcome, so it was practically impossible to apply meta-analysis because of follow-up variation, high ROB, and differences in intervention content. Thirdly, there were variations in health outcome measurements and pharmacists' interventions. Lastly, studies were primarily conducted in India, Pakistan, Nepal, and Sri Lanka included in the systematic review. Although RCTs were conducted in other South Asian countries, results may not be generalisable to all LMICs. Despite these limitations, we believe this review can help promote pharmacist-mediated care and pharmacy services in South Asia and thus improve patient outcomes.

## Conclusion

This systematic review underpins the contribution of pharmacists’ services in South Asian countries in terms of economic, clinical, and humanistic outcomes (ECHO). Interventions by the pharmacist have shown a positive impact on ECHO, but the impacts of their interventions on patients’ long-term health outcomes are yet to be explored in-depth, as most of the studies reported only the short-term outcomes. Therefore, future studies with appropriate study design, with randomisation in both interventional and control groups, are warranted to evaluate the pharmacist’s multi-dimensional roles on long-term outcomes in terms of economic (e.g., cost-effectiveness, cost-utility), clinical (e.g., improved health status), and humanistic (e.g., health-related quality of life) benefits. Also, a detailed pharmacoeconomic evaluation is required to make informed decision-making. Nevertheless, the findings of this review will be of particular interest to policymakers in countries where clinical pharmacy services are being newly implemented.
